# Development and Stepwise Optimization of Freeze‐Dried Seasoning Cubes From Traditional Fish Sauce: A Postharvest Approach for Value‐Added Aquatic Products

**DOI:** 10.1155/ijfo/2389589

**Published:** 2026-04-13

**Authors:** Van Thinh Pham, Nguyen Ngoc Thuy Duong Tran, Thanh Viet Nguyen

**Affiliations:** ^1^ Faculty of Tourism and Culinary, Ho Chi Minh City University of Industry and Trade, Ho Chi Minh City, Vietnam, huit.edu.vn; ^2^ Institute of Applied Technology and Sustainable Development, Nguyen Tat Thanh University, Ho Chi Minh City, Vietnam, ntt.edu.vn; ^3^ Center for Hi-Tech Development, Nguyen Tat Thanh University, Saigon Hi-Tech Park, Ho Chi Minh City, Vietnam, ntt.edu.vn

**Keywords:** fish sauce, freeze-drying, postharvest processing, seasoning cubes, value-added aquatic products

## Abstract

This study focuses on the technological development of freeze‐dried seasoning cubes from traditional Vietnamese fish sauce (60°N), aiming to improve postharvest stability and product versatility. Formulation trials were conducted by blending fish sauce with calamansi juice, garlic, chili, sugar, and maltodextrin to optimize flavor balance and structural integrity. Multistage freeze‐drying (10°C–50°C) was applied to evaluate the effects of drying temperature on moisture removal, structural properties, sensory quality, and retention of bioactive compounds. The optimal formulation (1:1 dilution, 10% calamansi, 15% garlic, 10% chili, 20% sugar, and 25% maltodextrin) combined with a final drying temperature of 50°C produced compact, well‐defined cube‐shaped products (approximately 25 × 25 × 8 mm) with a stable porous internal matrix and a smooth, nonsticky surface. The optimized cubes achieved a final moisture content of 2.79%, a sensory acceptance score of 4.50 ± 0.18, and retained considerable levels of bioactive compounds, including 44.64% DPPH radical scavenging activity, 21.15 mg GAE/100 g total polyphenols, and 24.75 mg AA/100 g vitamin C. These findings demonstrate a practical postharvest strategy to transform traditional liquid fish sauce into convenient, value‐added aquatic products suitable for broader culinary and export applications.

## 1. Introduction

Fish sauce is a traditional fermented condiment widely consumed across Southeast Asia and is increasingly gaining global culinary recognition. Produced through the enzymatic hydrolysis of fish proteins under high‐salt conditions, it is valued for its distinctive umami flavor and is recognized as a marine‐derived product rich in amino acids, peptides, and bioactive compounds [1, 2]. Beyond its culinary role, fish sauce represents a high‐value derivative of aquatic resources, and there is growing interest in transforming this traditional liquid condiment into modern, shelf‐stable seasoning formats to meet the increasing demands of global food service and export markets [3, 4].

However, the conventional liquid form of fish sauce presents several postharvest challenges, including limited storage stability, handling and transportation difficulties, and its strong aroma, all of which restrict its use in modern food processing systems and convenience products. These limitations highlight the need for alternative processing strategies and formulations that can improve both functionality and commercial applicability. Furthermore, transforming fish sauce from a liquid into a solid seasoning format presents a critical technological challenge. The solid‐state transformation must simultaneously preserve characteristic umami flavor, maintain structural integrity of the dried matrix, and retain sensitive bioactive compounds. Achieving an appropriate balance among flavor intensity, structural stability, and nutrient retention requires rational formulation design combined with controlled freeze‐drying parameters. This balance represents a key research problem that has not been sufficiently addressed in previous studies on fish sauce processing. Postharvest drying technologies provide effective means to enhance the stability and shelf life of perishable aquatic products. Among them, freeze‐drying has emerged as one of the most efficient preservation methods for retaining nutrients, flavors, and bioactive compounds while producing lightweight, shelf‐stable products [5, 6]. Compared with other drying techniques, freeze‐drying better preserves antioxidant capacity, sensory quality, and structural integrity [6, 7]. Freeze‐drying is particularly advantageous for high‐salt and high‐solute food systems, where thermal drying may accelerate chemical reactions and structural collapse. Reviews on freeze‐drying of complex food matrices have noted that low‐temperature dehydration helps maintain structural integrity and reduces quality deterioration in salt‐rich formulations [8, 9]. This is especially relevant for fish sauce, which contains high ionic strength and low‐molecular‐weight solutes. Unlike hot air drying, which relies on elevated temperatures that may accelerate Maillard reactions, salt‐induced protein denaturation, and oxidative degradation of aroma compounds, freeze‐drying operates under low‐temperature and vacuum conditions that minimize thermal stress. This distinction is particularly important for fish sauce, a high‐salt, high‐solute fermented system rich in low‐molecular‐weight peptides, free amino acids, and volatile aroma compounds that are highly sensitive to heat and oxidation. Moreover, spray‐drying, although efficient for powder production, typically involves atomization and rapid moisture removal at high inlet temperatures, which may cause partial volatilization of aroma‐active compounds and structural collapse in concentrated matrices. In contrast, freeze‐drying preserves the porous microstructure of the matrix, reduces chemical reaction rates, and better maintains both sensory and nutritional attributes in salt‐rich systems. Applying this technology to fish sauce offers new opportunities to develop innovative, convenient seasoning products such as tablets or cubes, which could enhance portability, facilitate culinary use, and expand market reach. Therefore, selecting an appropriate drying technology is essential for stabilizing fish sauce while preserving its distinctive sensory and nutritional properties. Previous studies have mainly focused on spray‐dried fish sauce powders or flavor enhancers derived from fish hydrolyzates [10, 11]. Aroma preservation is widely recognized as a critical quality factor in seasoning products. Drying methods involving high temperatures are known to cause volatilization and oxidative changes in aroma‐active compounds. Several reviews have emphasized that freeze‐drying better preserves aroma profiles compared with convective or spray‐drying due to minimized thermal stress and reduced oxidation [12, 13]. Although spray‐drying is cost‐effective and widely used, it often involves elevated inlet temperatures that can promote the loss of volatile aroma compounds and partial degradation of thermolabile nutrients. Several reviews have highlighted that flavor retention in spray‐dried systems strongly depends on carrier composition and process conditions, and significant aroma losses may occur during atomization and drying [14]. Reported rehydration times for spray‐dried seasoning powders typically range from 10 to 20 min depending on formulation and carrier systems, whereas freeze‐dried porous matrices generally rehydrate within a few minutes due to their open capillary structure [15]. In the present study, the developed freeze‐dried cubes achieved complete rehydration within approximately 3–5 min, further demonstrating their structural and functional advantages over spray‐dried powders. Additionally, freeze‐drying is generally recognized as superior for preserving volatile and heat‐sensitive compounds because its low‐temperature vacuum mechanism minimizes thermal degradation and oxidative reactions. Despite these advances, most previous studies have focused on spray‐dried fish sauce powders or flavor enhancers, while the development of structured seasoning formats such as freeze‐dried cubes remains largely unexplored. In particular, there is limited research integrating fish sauce with complementary ingredients and applying freeze‐drying to produce stable cube‐shaped seasoning products with preserved aroma and bioactive compounds. In contrast, limited research has addressed the development of integrated seasoning cubes combining fish sauce with complementary plant‐based ingredients such as garlic, chili, and citrus juice. These ingredients not only enrich flavor complexity but also supply bioactive compounds such as polyphenols and vitamin C, contributing to the functional and nutritional value of the final product. Moreover, proper packaging and storage remain essential to ensure quality preservation and consumer acceptance during distribution [16, 17]. In the context of global seafood trade, postharvest processing plays a critical role in improving product stability, reducing loss, and creating value‐added aquatic products. Fish sauce, as a unique fermented fish product from Southeast Asia, represents an important export‐oriented aquatic resource. Transforming traditional liquid fish sauce into shelf‐stable seasoning cubes through freeze‐drying offers a novel postharvest strategy to enhance convenience, extend shelf life, and diversify market applications, aligning with current trends in aquatic food processing and value‐added product development.

Therefore, this study aimed to develop and evaluate fish sauce‐based seasoning cubes using freeze‐drying technology and to identify practically optimal formulation and drying conditions through a stepwise formulation screening and process evaluation strategy. The research focused on sequentially screening formulation components (calamansi juice, garlic, chili, sugar, and maltodextrin) for sensory acceptability and structural integrity and evaluating the effect of drying temperature (10°C–50°C) on moisture removal, structural stability, and retention of bioactive compounds. This applied strategy was selected to support product development under realistic processing constraints rather than model‐based statistical optimization (e.g., response surface methodology [RSM]). From an industrial and commercial perspective, the use of premium‐grade fish sauce (60°N) inevitably increases raw material costs; however, it offers superior umami intensity, higher amino acid content, and improved sensory depth, which are critical for developing high‐value seasoning products. When combined with freeze‐drying technology, these sensory advantages may offset the increased material cost by enabling reduced dosage, extended shelf life, lower transportation weight, and access to premium domestic and export markets. Therefore, evaluating the trade‐off between cost and sensory performance is essential for assessing the commercial feasibility of freeze‐dried fish sauce seasoning cubes.

## 2. Materials and Methods

All experimental procedures were designed to evaluate postharvest processing strategies for transforming traditional fish sauce into value‐added seasoning cubes.

### 2.1. Materials

Premium fish sauce (Hanh Phuc brand, 60°N, Binh Dinh, Vietnam) was used as the primary raw material. Ly Son garlic (*Allium sativum* L.) was sourced from Quang Ngai Province, and Da Lat chili (*Capsicum annuum* L.) was obtained from Lam Dong Province, Vietnam. Fresh calamansi (*Citrus microcarpa* Bunge) fruits were purchased from a local market in Ho Chi Minh City. Granulated sugar (≥ 99.7% saccharose; Bien Hoa Sugar JSC, Vietnam) and maltodextrin (DE 10‐12; Roquette, Vietnam) were used as supplementary ingredients. Distilled water was prepared in the laboratory of Ho Chi Minh City University of Industry and Trade and used in all experiments.

### 2.2. Chemicals

All chemicals were of analytical grade. 2,2‐Diphenyl‐1‐picrylhydrazyl (DPPH, ≥ 95%, Sigma‐Aldrich, Steinheim, Germany) was used for antioxidant analysis. Hydrochloric acid (HCl, 35%–37%), methanol (≥ 99.7%), bromine, sodium carbonate (Na_2_CO_3_, ≥ 99%), sodium hydroxide (NaOH, ≥ 96%), sodium sulfite (Na_2_SO_3_, ≥ 98%), sulfuric acid (H_2_SO_4_, ≥ 96%), and thiourea (CH_4_N_2_S, ≥ 99%) were purchased from Merck (Darmstadt, Germany) and local suppliers in China and Vietnam. Folin–Ciocalteu reagent and sodium 1,2‐naphthoquinone‐4‐sulfonate (≥ 99%) were used for polyphenol determination. Whatman filter paper No. 1 (GE Healthcare, Buckinghamshire, UK) was employed in all filtration procedures.

### 2.3. Experimental Design

A stepwise formulation screening approach was employed, in which formulation components and freeze‐drying parameters were evaluated sequentially while keeping other variables constant. This approach was selected to facilitate applied product development and practical decision‐making for industrial‐oriented processing, rather than RSM‐based statistical optimization.

#### 2.3.1. Formulation Trials

Fish sauce (60°N) was blended with calamansi juice (5%–25%), garlic (5%–25%), chili (5%–25%), sugar (0%–30%), and maltodextrin (15%–30%). Formulations were evaluated for physicochemical properties and consumer acceptance. The optimal formulation was identified as 10% calamansi juice, 15% garlic, 10% chili, 20% sugar, and 25% maltodextrin, with fish sauce adjusted to 100%.

#### 2.3.2. Freeze‐Drying Conditions

Samples were prefrozen at −40°C for 12 h in a deep freezer prior to drying. Freeze‐drying was conducted using a freeze dryer (Model HR‐3, Harvest Right, Salt Lake City, UT, USA) equipped with a condenser temperature of −50°C and a vacuum pump (ultimate pressure < 0.05 mbar). During drying, the chamber pressure was maintained at < 0.1 mbar (operating pressure) as monitored by the system gauge. The effective chamber volume was approximately 5 L, with a condenser capacity of 3–4 kg of ice per 24 h. Samples were molded into cube‐shaped units with dimensions of approximately 25 × 25 × 8 mm. The relatively thin layer thickness (∼8 mm) was designed to minimize mass transfer resistance and promote uniform sublimation. Sample loading density was controlled to avoid overcrowding and ensure consistent heat and vapor transfer across trays during primary drying. The loading capacity was maintained at approximately 150 g per tray to ensure uniform heat transfer and vapor removal during sublimation. Controlling sample thickness and loading density was critical to avoid uneven drying and structural deformation. Drying was performed at shelf temperatures of 10, 20, 30, 40, and 50°C for up to 50 h under a controlled vacuum of < 0.1 mbar. The condenser temperature (−50°C) was maintained sufficiently lower than the product temperature to establish an effective vapor pressure gradient, facilitating efficient ice sublimation and vapor capture. This temperature differential is essential to prevent vapor backflow and ensure stable mass transfer during primary drying. The freeze‐drying process consisted of primary drying (ice sublimation under vacuum) followed by secondary drying to remove unfrozen bound water. Primary drying (ice sublimation) was performed under vacuum at the selected shelf temperature until the majority of free ice was removed, as indicated by the moisture–time curves. Secondary drying was subsequently continued by maintaining the same shelf temperature up to a total drying time of 50 h to reduce bound water and achieve the target final moisture (< 3%). Moisture content was periodically monitored to establish drying curves and assess drying kinetics. Among the tested conditions, 50°C for 50 h was selected as the optimal drying condition based on its ability to achieve final moisture content below 3% (in accordance with TCVN 7396:2004) while maintaining acceptable nutrient retention and structural integrity of the seasoning cubes.

#### 2.3.3. Experimental Flowchart

**FIGURE 1:**
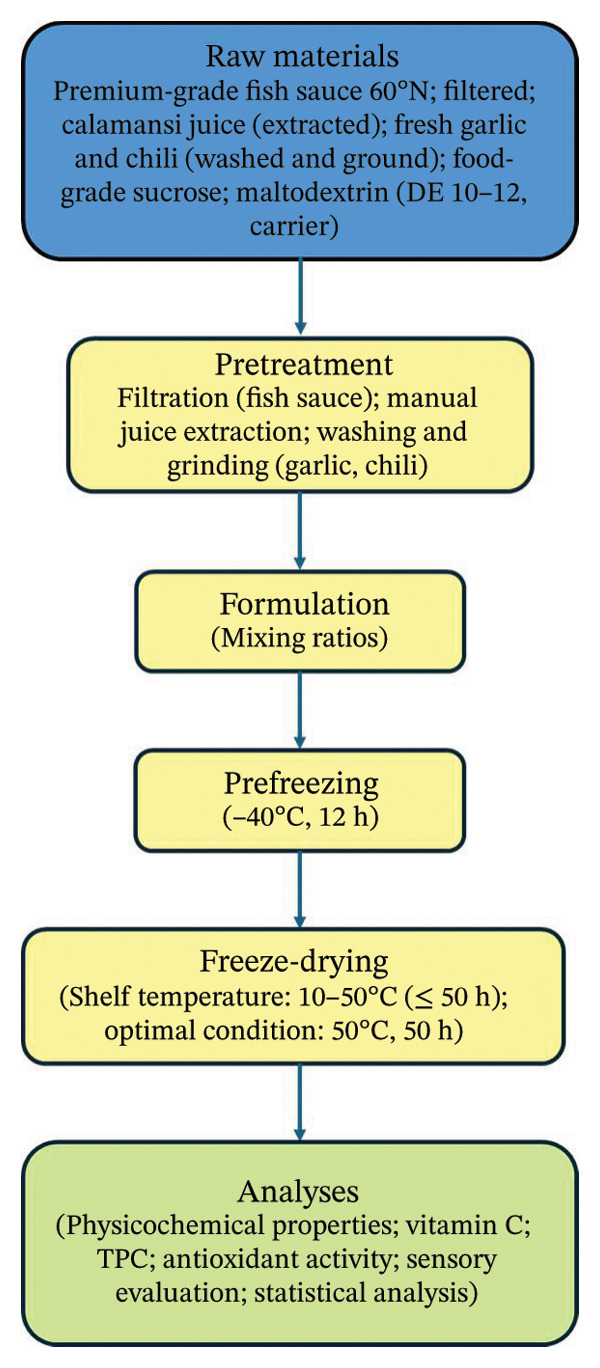
Experimental flowchart for the production and analysis of freeze‐dried fish sauce seasoning cubes.

The overall experimental procedure is summarized in Figure 1, illustrating the sequential steps from raw material preparation to formulation, prefreezing, freeze‐drying, and subsequent analyses.

### 2.4. Analytical Methods

#### 2.4.1. Salt Content

Salt content was determined using a digital salinity conductivity meter (Model B‐173, Horiba, Kyoto, Japan), calibrated with NaCl standards. Approximately 1 g of sample was dissolved in 50 mL of distilled water, and conductivity was measured at 25°C ± 1°C. Results were expressed as % NaCl (w/v) [4].

#### 2.4.2. Protein Content

Protein content was analyzed by the Kjeldahl method [4]. Samples (1 g) were digested with concentrated H_2_SO_4_ in the presence of a K_2_SO_4_–CuSO_4_ catalyst, distilled after alkalization with 40% NaOH, and titrated with standardized 0.1 N H_2_SO_4_. Total nitrogen was converted to crude protein using a factor of 6.25 [18].

#### 2.4.3. Moisture Content

Moisture content was determined according to TCVN 10788:2015 and AOAC 925.10. Ground samples (∼5 g) were dried in a hot‐air oven at 105°C until constant weight (mass variation < 0.005 g). Results were expressed as percentage moisture content (% w/w) [6].

#### 2.4.4. Vitamin C Content

Vitamin C was quantified using the spectrophotometric method of Ben Mussa & El Sharaa [19]. Absorbance was measured at 521 nm using a UV–Vis spectrophotometer (UV‐1900i, Shimadzu, Kyoto, Japan).

#### 2.4.5. Total Polyphenol Content

Total polyphenols were determined using the Folin–Ciocalteu method [6, 20]. Absorbance was measured at 765 nm using the same UV–Vis spectrophotometer. Results were expressed as mg gallic acid equivalents (GAE) per 100 g sample.

#### 2.4.6. Antioxidant Activity (DPPH Radical Scavenging)

The antioxidant activity was determined by the DPPH radical scavenging assay [21]. Absorbance was measured at 517 nm using the UV–Vis spectrophotometer. Radical scavenging activity was calculated as a percentage relative to the control.

### 2.5. Sensory Evaluation

Consumer acceptance was assessed using a 5‐point hedonic scale (1 = *dislike extremely*, 5 = *like extremely*), following standard sensory protocols [22]. Sixty untrained consumers, who were regular users of fish sauce or seasoning products, participated. The panel consisted of 28 males and 32 females aged between 20 and 45 years. Samples were coded and presented in randomized order using a Williams Latin square design. Evaluated attributes included color, aroma, sweetness, thickness, and overall acceptability. All participants were informed about the purpose of the study, and written informed consent was obtained prior to participation. The sensory evaluation was conducted in accordance with institutional guidelines.

### 2.6. Statistical Analysis

All experiments were performed in triplicate. Results were expressed as mean ± standard deviation (SD). Statistical analyses were conducted using JMP Pro 17 (SAS Institute Inc., Cary, NC, USA). One‐way ANOVA was applied to evaluate differences among treatments, followed by Tukey’s test (*p* < 0.05). Graphs were generated using Microsoft Excel 2021 (Microsoft Corp., Redmond, WA, USA).

## 3. Results and Discussion

### 3.1. Physicochemical Composition and Bioactive Properties of Raw Materials

The physicochemical and bioactive profiles of the main ingredients are summarized in Table 1. The premium fish sauce (60°N) exhibited very high total nitrogen (59.20 ± 1.35 g/L) and salinity (46,400 ± 210 mg/L), confirming its classification as high‐protein anchovy fish sauce according to Vietnamese standards (≥ 30°N). These values exceed those reported for Thai fish sauces (5–20 g/L total nitrogen) [4], reflecting extensive proteolysis during fermentation that generates umami‐active amino acids and peptides. Among the plant‐based ingredients, Ly Son garlic displayed the highest antioxidant activity (54.24% ± 2.11% DPPH), attributed to allicin and other sulfur compounds [23], and higher than typical common garlic varieties (35%–45%). This may relate to the unique geo‐climatic conditions of Ly Son Island. Da Lat chili showed the highest vitamin C content (30.14 ± 1.48 mg/100 g), consistent with reported ranges for fresh chilies [24, 25], making it a key contributor to both antioxidant activity and pungency. Calamansi contains lower vitamin C and polyphenol contents but contributes sourness and aroma [26]. Overall, combining high‐protein marine ingredients (fish sauce) with plant ingredients rich in antioxidants (garlic, chili, and calamansi) provides both a balanced sensory profile (salty‐sweet‐sour‐spicy‐aromatic) and enhanced functional properties, supporting the development of freeze‐dried fish sauce seasoning cubes as natural, bioactive‐rich condiments.

**TABLE 1:** Physicochemical and bioactive properties of the raw materials used in the formulation.

Parameter	Fish sauce 60°N	Ly Son garlic	Da Lat chili	Calamansi	Unit
Salinity	46400 ± 210	—	—	—	mg/L
Total nitrogen	59.20 ± 1.35	—	—	—	g/L
DPPH	—	54.24 ± 2.11	14.70 ± 0.84	19.39 ± 1.02	%
Total polyphenols	—	18.61 ± 0.92	17.68 ± 0.77	13.74 ± 0.68	mg GAE/100 g
Vitamin C	—	22.05 ± 1.15	30.14 ± 1.48	11.26 ± 0.56	mg AA/100 g

*Note:* Values are expressed as mean ± standard deviation (*n* = 3). DPPH: 2,2‐diphenyl‐1‐picrylhydrazyl radical scavenging activity; AA: ascorbic acid equivalents. “—” indicates that the parameter was not determined for that ingredient.

Abbreviation: GAE, gallic acid equivalents.

### 3.2. Effect of Formulation Factors on Sensory Attributes

The effects of formulation factors on sensory properties are shown in Figure 2 and 3. All factors significantly influenced at least one sensory attribute (*p* < 0.05), including saltiness, sourness, aroma, pungency, sweetness, spiciness, and viscosity. Dilution ratio: A 1:1 fish sauce‐to‐water ratio achieved the highest hedonic score (4.67 ± 0.12), balancing saltiness and flavor intensity. Lower ratios were overly salty, while higher ratios were perceived as weak [27]. Calamansi: The 10% level provided the most preferred sourness (4.93 ± 0.10), complementing the salty base without bitterness [28]. Garlic: 15% addition yielded the highest aroma pungency acceptance (4.10 ± 0.14), avoiding excessive sharpness at higher levels [29]. Chili: 10% was optimal (3.97 ± 0.09), providing balanced heat [30]. A formulation containing 20% sugar and 25% maltodextrin achieved the highest sweetness score (4.35) and viscosity (4.42) scores, producing a smooth texture similar to dipping sauces, whereas lower or higher levels were either bland or sticky [31]. This optimal formulation provided the most balanced sensory profile and overall consumer acceptance, establishing a scientific basis for developing value‐added fish sauce‐based seasoning products.

FIGURE 2Hedonic scores for seasoning formulations under different formulation factors: (a) dilution ratio; (b) calamansi concentration; (c) garlic level; (d) chili level. Bars represent mean ± SD (*n* = 60). Different letters (A–D) indicate significant differences (*p* < 0.05, Tukey’s test).

**Figure:**
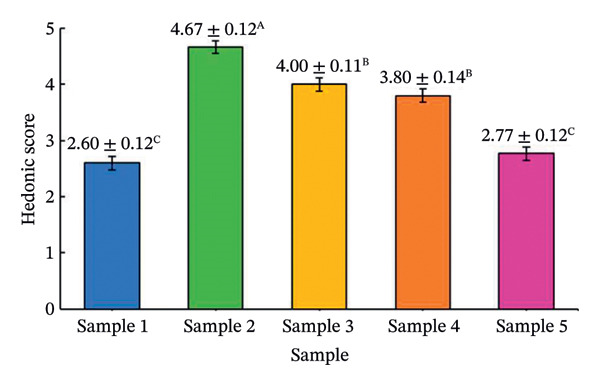
(a)

**Figure:**
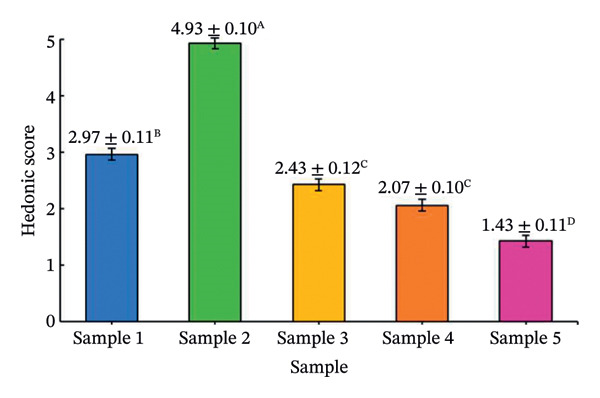
(b)

**Figure:**
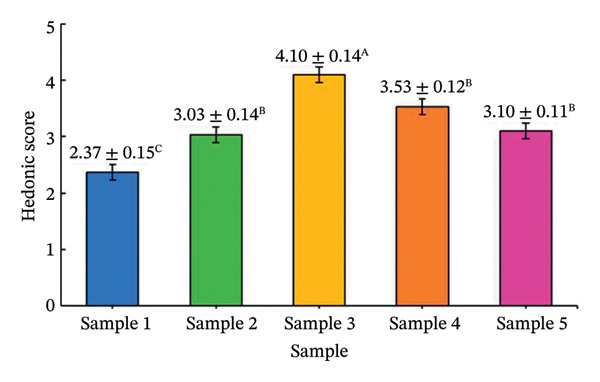
(c)

**Figure:**
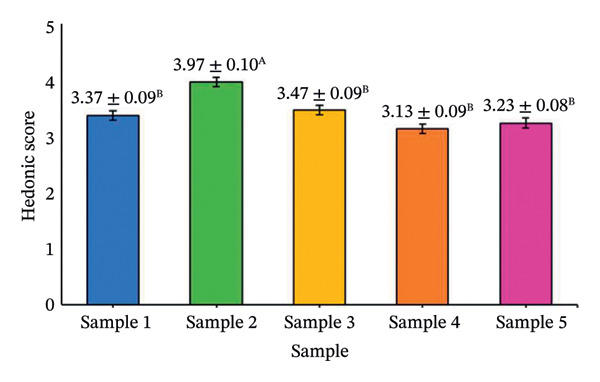
(d)

FIGURE 3Hedonic scores for (a) sweetness preference and (b) viscosity preference among 16 sugar–maltodextrin formulations. Values are expressed as mean ± SD (*n* = 30).

**Figure:**
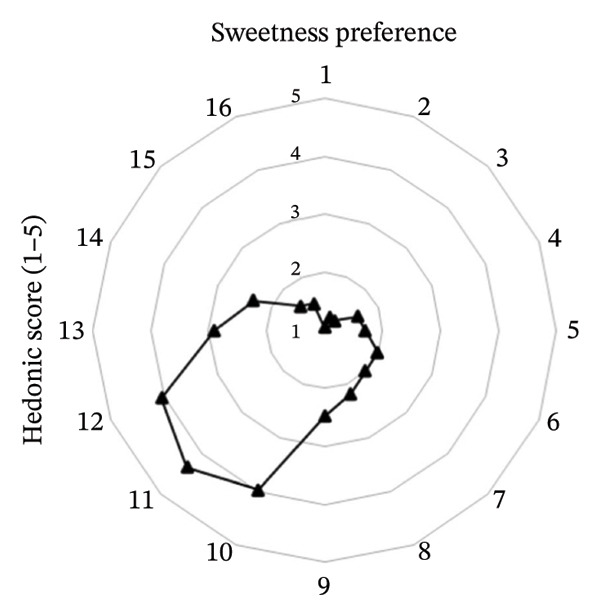
(a)

**Figure:**
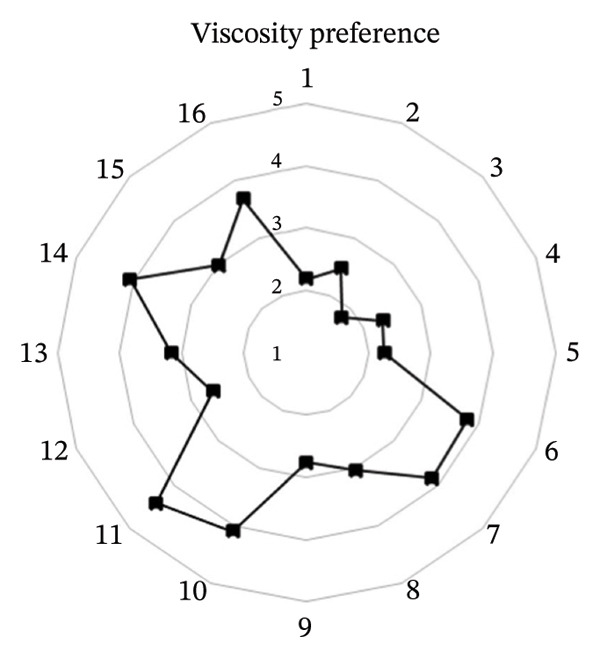
(b)

### 3.3. Effect of Drying Temperature on Product Quality

#### 3.3.1. Structural Characteristics and Rehydration Properties

The structural changes of selected sugar–maltodextrin formulations during stepwise drying at different temperatures (20°C–50°C) are presented in Table 2.

**TABLE 2:** Structural changes of selected sugar–maltodextrin formulations during stepwise drying at 20°C–50°C.

Temp (°C)	Sugar/maltodextrin (%)	Representative sample			Structural observation
20	10/15–25				Hollow, puffed, partially cohesive
					

30	20/15–25				25% maltodextrin, smooth and uniform

40	20/20–25				25% maltodextrin, no puffing

50	20/25				Compact, porous structure, easy demolding

*Note:* Formulations with stable geometry at 10°C were selected for progressive drying at increasing temperatures. Table 2 summarizes visible structural characteristics (cohesiveness, puffing, and hollowness) observed after each drying stage, highlighting the formulation that maintained optimal structural integrity across all temperatures. The final optimal drying condition (50°C, 20/25) is highlighted.

Following the initial screening at 10°C (Table 3), the structural evolution of selected sugar–maltodextrin formulations during stepwise drying at increasing temperatures (20°C–50°C) is further analyzed. At 10°C, formulations were screened for their ability to retain shape during primary drying. Only formulations that maintained stable geometry were advanced. Increasing the drying temperature from 20°C to 40°C progressively improved surface smoothness and internal structure, particularly at 25% maltodextrin. At 50°C, the 20% sugar + 25% maltodextrin formulation produced compact, porous cubes that demolded easily without deformation (Figure 4). Maltodextrin possesses a relatively high glass transition temperature (Tg), contributing to increased matrix rigidity and reduced molecular mobility during dehydration. In contrast, simple sugars exhibit lower Tg values and act as plasticizing agents, potentially lowering system Tg and increasing the risk of structural collapse when present in excess. Moreover, maltodextrin increases total solid content and elevates collapse temperature (Tc), further stabilizing the matrix during sublimation and secondary drying. Therefore, balancing maltodextrin and sugar proportions enables modulation of the overall glass transition behavior, ensuring structural stability while maintaining desirable sensory attributes [32]. The glass transition behavior of multicomponent systems such as sugar–maltodextrin mixtures follows a compositional dependence, where the overall Tg of the matrix is influenced by the relative proportions of high‐Tg polymers and low‐Tg plasticizing sugars. Increasing the maltodextrin fraction raises the composite Tg and collapse temperature, thereby enhancing structural rigidity during primary and secondary drying [33]. Conversely, excessive simple sugars may depress the system Tg due to their plasticizing effect, increasing molecular mobility and the likelihood of structural deformation at elevated shelf temperatures [34]. Therefore, the selected maltodextrin‐to‐sugar ratio (25% maltodextrin and 20% sugar in the optimal formulation) represents a balance that maintains the matrix Tg above the processing temperature while preserving desirable sensory sweetness and flavor characteristics.

**TABLE 3:** Screening of sugar–maltodextrin formulations at 10°C drying temperature.

Sugar (%)	Representative samples				Structural observation
	15%	20%	25%	30%	
0					Hollow or unstable structure at all maltodextrin levels

10					Improved integrity with increasing maltodextrin

20					Moderate puffing at low maltodextrin; stable at ≥ 25%

30					Collapse or excessive puffing at all maltodextrin levels

*Note:* Sixteen formulations combining sugar (0%–30%) and maltodextrin (15%–30%) were screened at 10°C for structural stability after freezing and primary drying. Formulation codes (1–16) correspond to sugar–maltodextrin ratios used in subsequent drying trials. The optimal formulation selected for subsequent drying trials is highlighted with a red frame for clarity.

FIGURE 4Flat surface and cross‐sectional morphology of the optimized seasoning cube after freeze‐drying. (a) The flat surface view shows a dense, cohesive structure with minimal deformation. (b) Cross‐sectional view reveals a uniform internal structure with low porosity and no puffing, indicating good structural stability during drying.

**Figure:**
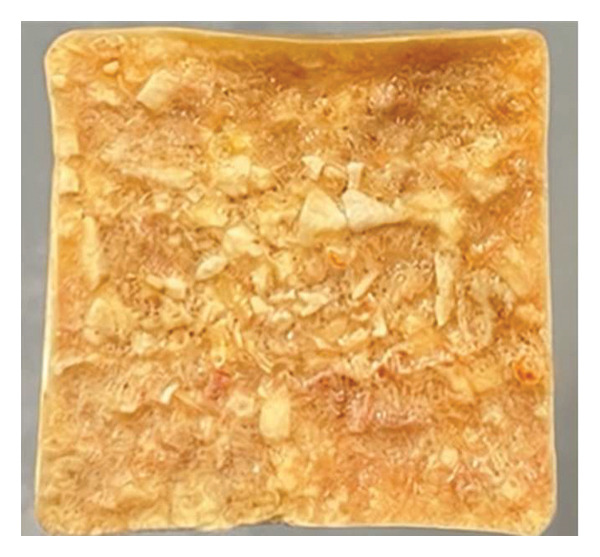
(a)

**Figure:**
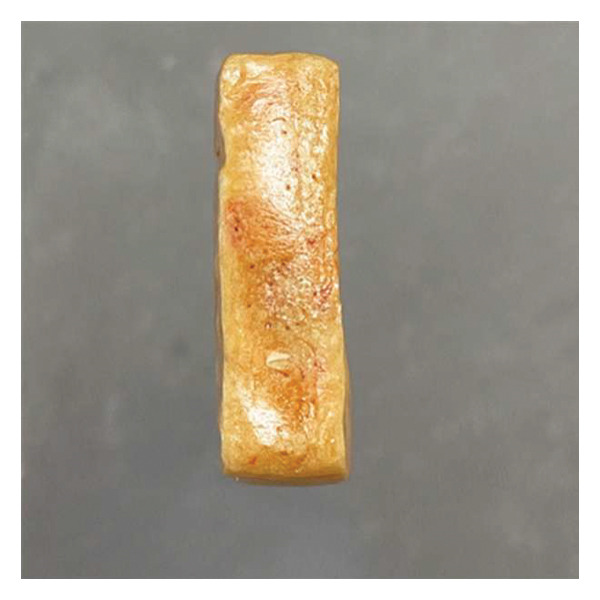
(b)

**FIGURE 5:**
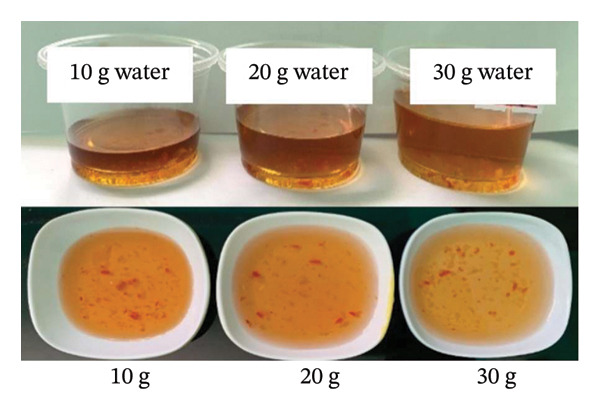
Rehydration appearance of seasoning cubes with varying water volumes.

**FIGURE 6:**
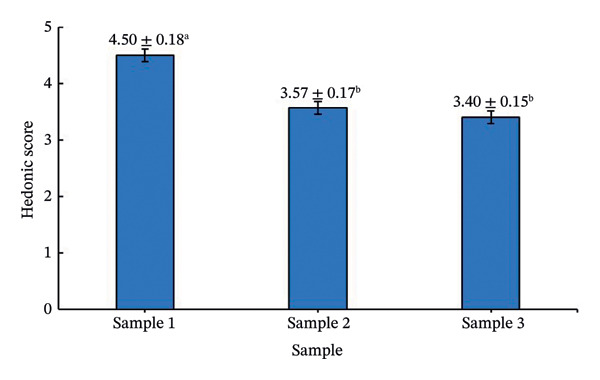
Hedonic scores of rehydrated seasoning cubes prepared with different water volumes. Sample 1: 10 g water; Sample 2: 20 g water; Sample 3: 30 g water. Values are expressed as mean ± SD (*n* = 30). Different letters (a‐b) above the bars indicate significant differences between samples (*p* < 0.05, Tukey’s multiple range test).

This behavior reflects the stabilizing role of maltodextrin in forming a three‐dimensional network that prevents puffing and collapse during sublimation, while sugar influences ice crystallization and stickiness. The stabilizing effect of maltodextrin may be attributed to its ability to increase solid content and promote the formation of an amorphous glassy matrix during freeze‐drying, thereby enhancing matrix rigidity and reducing structural collapse and excessive puffing. These phenomena are consistent with previous observations in freeze‐dried fruit purées and dairy matrices [35, 36]. Rehydration behavior is shown in Figure 5. Cubes rehydrated with 10 g of water displayed the best appearance and flavor, confirmed by sensory scores (Figure 6). Higher rehydration volumes produced diluted taste and lower preference.

The superior structural integrity, controlled rehydration behavior, and high sensory scores observed in this study highlight the technological advantages of freeze‐drying compared with conventional drying approaches for seasoning products. Spray‐dried fish sauce powders reported in previous studies are typically produced at inlet air temperatures ranging from 150°C to 180°C, which may lead to partial degradation of volatile aroma compounds and thermolabile nutrients [10]. In addition, high carrier incorporation levels (commonly 40%–60% maltodextrin) are often required to reduce stickiness and improve powder flowability during spray drying [37]. Such conditions may dilute flavor intensity and influence the sensory quality of the final product. In contrast, the freeze‐dried seasoning cubes developed in this study achieved structural stability at lower carrier ratios (15%–30%), benefiting from the formation of a porous sublimation matrix under low‐temperature conditions. The optimized cubes exhibited high hedonic scores (4.50 ± 0.18) and maintained desirable rehydration clarity, suggesting improved flavor preservation compared with spray‐dried counterparts reported in the literature. The porous microstructure generated during freeze‐drying also facilitated controlled dissolution, producing a reconstituted broth with sensory characteristics closer to traditional liquid fish sauce. Previous studies have similarly reported that freeze‐drying can better preserve aroma compounds and structural integrity in fermented seasoning products due to the low‐temperature sublimation process [38]. These findings are consistent with the present results, in which the freeze‐dried fish sauce seasoning cubes maintained structural stability, required lower carrier levels, and exhibited favorable rehydration and sensory properties. Overall, these comparisons support the suitability of freeze‐drying as an effective processing strategy for developing high‐quality, value‐added seasoning products. These findings demonstrate that the formulation design and matrix composition strongly influence the structural stability of the seasoning cubes during freeze‐drying. The structural integrity established at this stage also plays a critical role in determining drying kinetics and moisture removal behavior at different drying temperatures, which are discussed in the following section.

#### 3.3.2. Effect of Drying Temperature on Moisture Content

**FIGURE 7:**
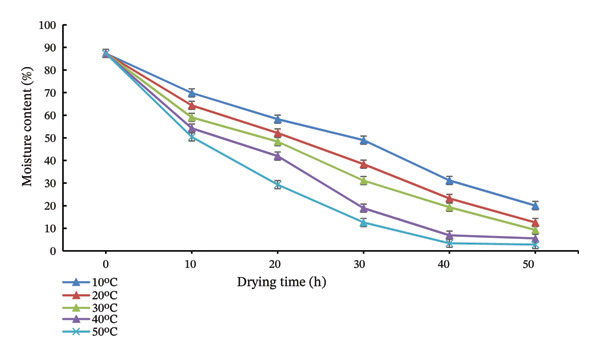
Effect of drying temperature on the moisture content of freeze‐dried seasoning cubes during freeze‐drying. Higher drying temperatures (40°C–50°C) accelerated moisture removal, resulting in final moisture contents below 3% after 50 h, whereas samples dried at 10°C retained significantly higher moisture throughout the drying process.

Drying temperature significantly affected moisture reduction (Figure 7). Higher temperatures (40°C–50°C) accelerated water removal, achieving a final moisture content of 2.79% at 50°C, meeting TCVN 7396:2004 standards, while lower temperatures failed to reach this level within 50 h. This is explained by increased vapor pressure gradients and faster sublimation at higher temperatures, consistent with Nowak & Jakubczyk [8]. The drying curves exhibited an initial constant‐rate period followed by a falling‐rate phase, with critical moisture reached earlier at higher temperatures.

#### 3.3.3. Effect of Drying Temperature on Antioxidant Activity, Polyphenols, and Vitamin C

Drying temperature significantly influenced DPPH radical scavenging activity, total polyphenols, and vitamin C (Figure 8).

FIGURE 8Effect of drying temperature on (a) DPPH radical‐scavenging activity, (b) vitamin C content, and (c) total polyphenol content of seasoning cubes. Values are means ± SD (*n* = 3). Different letters (A–E) indicate significant differences among temperatures (*p* < 0.05, Tukey’s test).

**Figure:**
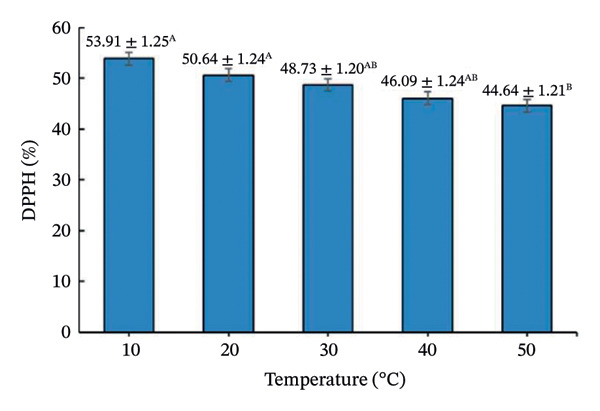
(a)

**Figure:**
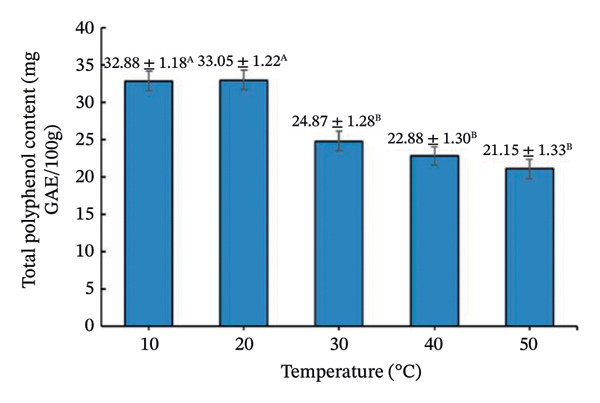
(b)

**Figure:**
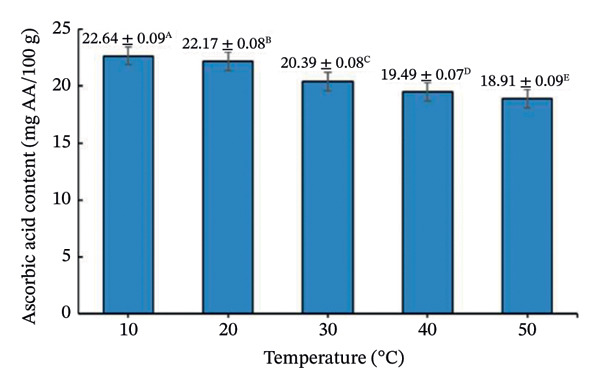
(c)

DPPH activity was highest at 10°C (53.91 ± 1.24%) and declined with increasing temperature. Polyphenols peaked at 20°C (33.05 ± 1.30 mg GAE/100 g) and then decreased, while vitamin C showed maximum retention at 10°C and declined progressively. These patterns reflect thermo‐oxidative degradation of phenolics and vitamin C during drying [39–41], following Arrhenius‐type kinetics [42]. Overall, lower temperatures favor better preservation of bioactive compounds but prolong drying time, whereas higher temperatures increase efficiency but cause losses. This trade‐off is typical in aquatic product drying systems [43].

#### 3.3.4. Optimal Drying Temperature Selection

It should be noted that the present work adopted a stepwise screening and process evaluation strategy rather than a full factorial or RSM‐based optimization and therefore does not model factor interactions statistically. Nevertheless, the sequential approach effectively identified practically optimal conditions for product development under realistic processing constraints. From a food physics perspective, the structural stability of the freeze‐dried cubes at elevated secondary drying temperatures can be attributed to the glass transition behavior of the amorphous matrix. In high‐salt, high‐sugar systems, drying above the glass transition temperature (Tg) typically increases the risk of matrix collapse due to reduced molecular viscosity and increased mobility. However, the incorporation of maltodextrin increased the total solid content and effectively elevated the Tg of the formulation, allowing the matrix to remain in a glassy, mechanically stable state during secondary drying at 50°C. In addition to its carrier function, maltodextrin played a critical structural role by increasing matrix viscosity, reducing stickiness, and limiting molecular mobility during drying. This effect mitigated structural collapse and excessive puffing commonly observed in high‐sugar or high‐salt systems subjected to elevated drying temperatures. Consequently, the freeze‐dried cubes maintained their compact structure despite secondary drying at 50°C. Nevertheless, future work may apply RSM or factorial designs to quantify interaction effects and further refine the optimization for scale‐up. From a process perspective, although lower secondary drying temperatures favored higher antioxidant retention, they resulted in prolonged drying time and increased energy consumption per batch. In contrast, drying at 50°C provided a balanced compromise by significantly enhancing drying kinetics and ensuring moisture compliance (< 3%) while maintaining bioactive retention at levels sufficient to preserve sensory quality and functional value. Therefore, the selection of drying temperature was based not solely on moisture reduction efficiency but on an integrated assessment of drying kinetics, structural stability, and bioactive retention. Among tested conditions, 50°C provided the best balance between moisture reduction, structure, and bioactive retention, achieving 2.79% moisture, compact structure, and acceptable antioxidant levels (44.64% DPPH, 21.15 mg GAE/100 g polyphenols, 24.75 mg AA/100 g vitamin C). Although some degradation occurred, these values are adequate for maintaining sensory and functional quality. The final physicochemical and bioactive properties of the optimized cubes are summarized in Table 4.

**TABLE 4:** Physicochemical and bioactive properties of the optimized freeze‐dried fish sauce seasoning cubes (*n* = 3).

Parameter	Mean ± SD (*n* = 3)	Unit
Moisture	2.79 ± 0.05	%
DPPH radical scavenging activity	44.64 ± 1.24	%
Total polyphenols	21.15 ± 1.30	mg GAE/100 g
Vitamin C	24.75 ± 0.08	mg AA/100 g

Similar approaches have been reported for spray‐dried fish sauce powders and shrimp hydrolyzate flavoring products [10, 11], but few studies have integrated fish sauce with plant ingredients into compact freeze‐dried cubes. This study fills that gap and provides practical insights for postharvest processing of aquatic condiments. Compared with conventional spray‐dried fish sauce powders, the freeze‐dried cube format provides superior structural integrity and dosage precision, while maintaining comparable levels of antioxidant activity, highlighting its suitability for high‐value postharvest aquatic products. From an industrial feasibility standpoint, the selection of 50°C also reduces overall processing time and energy consumption per batch, which is particularly relevant when using premium‐grade fish sauce as a raw material. Faster secondary drying improves production throughput and partially compensates for higher ingredient costs, thereby enhancing economic viability. This balance between processing efficiency, product quality, and raw material value is critical for industrial‐scale adoption.

## 4. Conclusion

This study developed and optimized a freeze‐drying strategy to transform traditional Vietnamese fish sauce into compact, shelf‐stable seasoning cubes through formulation optimization and controlled multistage drying. The optimal combination of ingredients and drying temperature produced cubes with desirable structural characteristics, extended shelf life, and good retention of sensory and functional properties. The findings highlight freeze‐drying as an effective postharvest processing technology for converting liquid aquatic condiments into value‐added seasoning formats suitable for modern culinary applications. This approach addresses key challenges related to storage, transportation, and aroma handling of fish sauce products while maintaining the distinctive sensory and nutritional characteristics of fish sauce. This optimized postharvest strategy provides a practical pathway for diversifying and internationalizing aquatic food products, supporting both domestic utilization and export‐oriented product development within the seafood sector. From an industrial perspective, the developed freeze‐dried seasoning cubes offer advantages in terms of reduced transportation weight, improved storage stability, and easier dosage control compared with conventional liquid fish sauce. This product format demonstrates strong potential for large‐scale production and commercialization in food service, instant food, and export‐oriented markets. Although the use of premium‐grade fish sauce increases raw material costs, the resulting improvements in sensory quality, flavor concentration, and product stability enable reduced usage levels and justify positioning the product in high‐value market segments. When combined with the logistical advantages of freeze‐dried cubes, including lower transportation costs and extended shelf life, the overall cost–benefit balance supports the commercial potential of this product. Notwithstanding these promising findings, several methodological and practical limitations should be acknowledged. The study was conducted at laboratory scale, and scale‐up performance under industrial freeze‐drying systems may involve additional process variability. Furthermore, the economic assessment was based on conceptual cost–benefit considerations rather than detailed techno‐economic modeling. Long‐term storage stability under diverse packaging and distribution conditions was also beyond the scope of this work. Future research should therefore expand both the technological and functional scope of freeze‐dried fish sauce seasoning cubes. Subsequent studies may focus on pilot‐scale validation, comprehensive shelf‐life modeling, and full economic feasibility analyses to support industrial commercialization. In addition, the development of reduced‐sodium formulations (e.g., 20%–30% salt reduction) could align the product with global healthy food trends while maintaining umami intensity and structural stability. The incorporation of functional ingredients such as probiotics, bioactive peptides, or natural antioxidants may further enhance nutritional and functional value, opening new opportunities for multifunctional seasoning systems in value‐added aquatic product markets.

## Author Contributions

Van Thinh Pham: conceptualization, methodology, validation, formal analysis, investigation, data curation, and writing–original draft. Nguyen Ngoc Thuy Duong Tran: methodology, validation, formal analysis, and visualization. Thanh Viet Nguyen: conceptualization, methodology, validation, formal analysis, investigation, data curation, writing–original draft, visualization, supervision, project administration, funding acquisition, and writing–review and editing.

## Funding

No funding was received for this research.

## Conflicts of Interest

The authors declare no conflicts of interest.

## Data Availability

All data generated or analyzed during this study are included in this published article. Additional raw data are available from the corresponding author upon reasonable request.
